# Obesity Inequalities According to Place of Birth: The Role of Education

**DOI:** 10.3390/ijerph15081620

**Published:** 2018-07-31

**Authors:** Elena Rodriguez-Alvarez, Nerea Lanborena, Luisa N. Borrell

**Affiliations:** 1 Department of Nursing I, University of the Basque Country (UPV/EHU), 48940 Leioa, Spain; nerea.lamborena@ehu.eus; 2 OPIK-Research Group for Social Determinants of Health and Demographic Change, University of the Basque Country (UPV/EHU), 48940 Leioa, Spain; 3 Department of Epidemiology & Biostatistics, Graduate School of Public Health & Health Policy, City University of New York, New York, NY 10027, USA; Luisa.Borrell@sph.cuny.edu

**Keywords:** obesity, immigration, education, inequalities, health survey

## Abstract

This study examined obesity inequalities according to place of birth and educational attainment in men and in women in Spain. A cross-sectional study was conducted using data from the Spanish National Health Survey 2011–2012 and from the European Health Survey in Spain 2014. We used data for 27,720 adults aged 18–64 years of whom 2431 were immigrants. We used log-binomial regression to quantify the association of place of birth with obesity before and after adjusting for the selected characteristics in women and in men. We found a greater probability of obesity in immigrant women (PR: 1.42; 95% CI: 1.22–1.64) and a lower probability of obesity in immigrant men (PR: 0.73; 95% CI: 0.59–0.89) relative to natives after adjustment. Significant heterogeneity was observed for the association of place of birth and obesity according to education in men (*p*-interactions = 0.002): Men with lower educational levels (PR: 0.47; 95% CI: 0.26–0.83) have a protective effect against obesity compared with their native counterparts. This study suggests that place of birth may affect obesity in women and in men. However, this effect may be compounded with education differently for women and men.

## 1. Introduction

Obesity currently represents a world-wide public health problem, given its impact on chronic diseases and premature mortality [1,2]. In addition, obesity is associated with a high financial burden for health care utilization [3]. Over the past four decades, the global prevalence of obesity in adults has tripled in men (3.2% to 10.8%) and doubled in women (6.4% to 14.9%). Projections for 2025 indicate that the overall prevalence of obesity will reach 18% in men and exceeds 21% in women [4]. Currently, more than half of adults are overweight and almost one in six adults in Europe is obese [5]. It is estimated that a 5% reduction in body mass index (BMI) in the European population would entail a 16.7% decrease in the prevalence of obesity related-diseases such cancer, stroke, and type 2 diabetes [6].

In recent years, extant evidence suggests that health status is conditioned by the unequal distribution of social determinants [7,8]. For instance, place of birth and socioeconomic position may explain inequalities in risks associated with health and/or disease, as a result of the different opportunities and resources people may have to achieve good health [9]. In fact, place of birth and the migratory process from the least developed countries to those of greater economic development, such as the countries of the European Union with a very high Human Development Index (HDI), represent a crucial factor for the study of social inequalities in health [10]. Therefore, most studies conclude that the worst socioeconomic and labor conditions, the lowest social support and the highest discrimination of the immigrant population may explain their poor mental and physical health outcomes compared to the native population [11,12,13]. With regards to obesity in Europe, studies report higher prevalence estimates of obesity among immigrant population compared to their native counterparts [14,15,16,17]. However, this difference may be explained by the lower socioeconomic position of the immigrant population. In contrast, European studies on native population only show that educational attainment, as an indicator of socioeconomic position, is the main determinant in obesity inequalities, exhibiting a clear direct social gradient, i.e., higher prevalence of obesity with lower education attainment [18,19,20]. However, and in contrast to the USA, where the role of education on immigrants’ health depends on gender and country of birth [21,22], no studies have examined the effect of place of birth and education together on obesity inequalities in European men and women.

Spain is one of the countries with the highest prevalence of obesity (17% in men and 16% in women) in the European Union and among countries presenting greater obesity inequalities among the native population by educational attainment, especially among women [18,20,23]. Moreover, Spain is the fourth country in the European Union when it comes to the proportion of immigrants (13.6% in 2017) [24]. Specifically, during the last two decades, Spain has experienced an intense growth of the immigrant population from less developed countries for economic reasons. Most immigrants are coming from Latin America (38%; mainly from Ecuador, Colombia, Peru and Bolivia) Maghreb (14%; mainly from Morocco), sub-Saharan Africa (4%; mainly Senegal and Nigeria), Asia (7%; mainly China) and the rest of non-very high developed European Union countries (10%; mainly Romania). However, and despite this trend as well as the importance of place of birth and the migratory process as determinants of inequality [10], few studies have focused on obesity inequalities between immigrant and native populations in Spain [25,26]. Furthermore, the immigrant population usually has a lower level of education than the native population [27]. Therefore, this study aimed to examine (a) obesity inequalities between immigrant and native populations; and (b) the combined effect of place of birth and educational attainment in men and in women in Spain for the years 2011–2014.

## 2. Materials and Methods

### 2.1. Data Source and Study Population

The study was based on a cross-sectional analysis of data obtained from the Spanish National Health Survey (SNHS) of 2011–2012 and from the European Health Survey in Spain (EHSS) of 2014 conducted by the Ministry of Health, Social Services and Equality in collaboration with the National Institute of Statistics. The surveys used representative samples of the non-institutionalized population in Spain through a stratified multistage sampling. More detailed information on the methodology of these surveys has been described elsewhere [23]. Analyses were based on information obtained from the individual questionnaires conducted in selected households, with a sample of 21,007 adults for the 2011–2012 SNHS and 22,842 adults for the 2014 EHSS. The response rates were 71% for both surveys. For the 43,849 people who completed the individual questionnaires, we excluded records of individuals under 18 years of age (*n* = 941) and older than 64 years (*n* = 12,416), those born in countries with an HDI considered as very high in 2015 (>0.80; *n* = 738), and those missing info on BMI (*n* = 1105), occupation (*n* = 570) and other covariates (*n* = 359). These exclusions yielded an analytical sample of 27,720 of whom 2431 were immigrants. This study used publicly available data obtained from the Ministry of Health Social Services and Equity in Spain. Thus, ethical approval for the study was not required.

### 2.2. Variables

The dependent variable was obesity, defined according to the World Health Organization (WHO) as BMI ≥ 30 kg/m^2^, and calculated using self-reported weight and height [28]. The independent variable was place of birth categorized as native for those born in Spain, and immigrants for those born in a country with non-very high HDI in 2015 (Europe, Africa, Latin America and Asia). In addition, educational attainment was considered as an effect measure modifier and was categorized as primary education or less, secondary and graduate or higher education [25].

Consistent with previous studies, we included the following covariates as potential confounders: age (18–24, 25–44 and 45–64 years) [26], living arrangement (married/couple and other), employment (employed, unemployed and other) and social class (using the classification of the Spanish Society of Epidemiology as non-manual workers for class I, II and III and manual workers for class IV and V) [29]. Consistent with a previous study [30], self-reported health responses were aggregated as good (very good and good) and poor (fair, bad and very bad). Finally, for health behaviors, we included smoking (current, former and never smoker) consumption of alcohol (frequent, occasional and not last year/never), physical activity at work and at leisure time (active and sedentary) and daily consumption of fruits and vegetables (yes/no) [25].

### 2.3. Statistical Analysis

Descriptive statistics for selected characteristics were calculated for the total population and according to place of birth in women and in men. In addition, prevalence estimates for obesity were calculated for each covariate according to place of birth in women and in men. Chi-square of independence and Cochran-Mantel-Haenszel statistics were used to assess significance associations between each covariate and (1) place of birth, and (2) place of birth and obesity in women and in men. Similarly, *t*-tests were used to assess differences for age and BMI according to place of birth in women and in men. We used log-binomial regression to quantify the association of place of birth with obesity in women and men before and after controlling for selected covariates. We tested interactions terms between place of birth and educational attainment in the fully-adjusted model for women and for men.

Data management procedures were carried out using SPSS 24.0. (IBM, Armonk, NY, USA) whereas the statistical analyses were conducted using SUDAAN 11.0.1 (RTI, Research Triangle Park, NC, USA) to take into account the complex sampling design and yield unbiased standard error estimates. Sample sizes presented in Table 1 were a-weighted, but all other estimates (proportions, standard errors, prevalence ratios [PR] and their 95% confidence intervals (95% CI) were weighted.

## 3. Results

Table 1 shows the distribution of selected sociodemographic, health status and health behavior characteristics for immigrant and native women and men. Around half of the immigrant population came from Latin America (54% men and 45% women). Immigrant women were younger, less educated, more likely to be employed as manual workers, have a partner, and rate their health status as worse than native women (all *p*-values < 0.001). In relation to health behaviors, immigrant women were less likely to be smokers, consume alcohol, be sedentary at work but more likely to have leisure time than their native counterparts (all *p*-values < 0.01). There was no association between consumption of fruits and vegetables and place of birth in women. As with women, immigrant men were younger, less educated, more likely to be employed as manual workers and to have a partner than their native counterparts (all *p*-values < 0.001). Immigrant men were also less likely to smoke, consume alcohol, be sedentary at work but more likely to have leisure time than native men (*p*-values < 0.01).

Table 2 presents the prevalence estimates for obesity for selected characteristics according to place of birth in men and in women. For women, the prevalence of obesity was higher in immigrants (20%) than in native women (12.5%), with the highest prevalence among those from Africa (30.9%). When compared to native women, older age, low educational attainment, manual social class, married or living couple, and poor perceived health were associated with higher prevalence estimates of obesity among immigrants (all *p*-values < 0.001). In addition, lower prevalence of obesity was associated with smoking status, alcohol consumption, and physical activity at the work place and leisure in immigrant women relative to their native counterparts (all *p*-values < 0.05).

In men, the prevalence of obesity was lower among immigrants relative to their native peers (12.5% vs. 16.9%; *p* < 0.001), especially among those coming from Asia (6.1%). Immigrant men exhibited lower prevalence estimates for obesity associated with age, employment status, living arrangement, self-rated health, smoking status and leisure-time physical activity compared with native men (all *p*-values < 0.05). Moreover, low educational attainment and manual social class were associated with lower prevalence of obesity among immigrant men whereas the opposite was true for native men (*p*-values < 0.001).

Table 3 shows unadjusted and adjusted prevalence ratios for obesity with their 95% confidence intervals (CI) for immigrant men and women relative to their native counterparts. For women, the probability of obesity was 1.60 (95% CI: 1.38–1.86) times higher in immigrants compared with natives. This probability increased after adjusting for age and self-rated health (PR: 1.73; 95% CI: 1.50–2.00). However, the association while attenuated remained significant in the fully-adjusted model (Model 5: PR: 1.42; 95% CI: 1.22–1.64). For men, the probability of obesity was 26% (PR: 0.74; 95% CI: 0.60–0.89) lower in immigrants relative to natives. This lower probability of obesity remained significant and nearly unchanged in the final model (PR: 0.73; 95% CI: 0.59–0.89).

**Figure 1:**
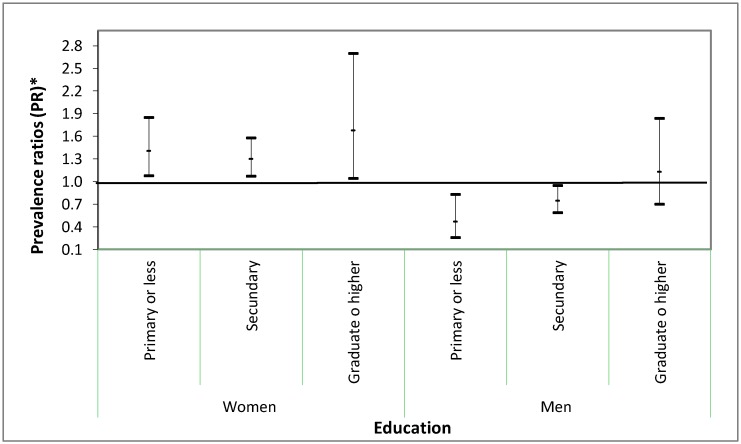
Prevalence ratios (PR) * and 95% confidence intervals of obesity for place of birth according to educational attainment in women and in men: Spanish National Health Survey 2011–2012, and European Health Survey in Spain 2014. * Adjusted for survey year, age, self-rated health, employment status, living arrangement, physical activity at work and at leisure time, smoking, alcohol consumption, daily consumption of fruits and vegetables and social class.

Heterogeneity for the association between place of birth and obesity was observed according to education in men (*p*-interaction = 0.002) but not in women (*p*-interaction = 0.94). Figure 1 shows that immigrant men with at least a primary (PR: 0.47; 95% CI: 0.26–0.83) and secondary (PR: 0.75; 95% CI: 0.59–0.95) education have a protective effect against obesity compared with their native counterparts. Immigrant women have higher probability of obesity than native women regardless of educational attainment. However, the probability of obesity was greater among immigrant women with a graduate or higher education.

## 4. Discussion

We found a greater probability of obesity in immigrant women and a lower probability of obesity in immigrant men relative to their native counterparts, after adjusting for sociodemographic characteristics, self-rated health and health behaviors. In addition, this association depends on education in men with those with lower educational levels having a lower probability of obesity. Although we did not find heterogeneity of the association between place of birth and obesity according to education among women, immigrant women were more likely to be obese than the native regardless of their education, with the higher probability observed among women with the highest education.

For men, the finding of lower prevalence of obesity among immigrants from Africa, Asia and Latin America, relative to natives is consistent with studies conducted in Spain and in other countries of southern Europe [17,26,31]. However, the obesity inequalities between immigrants and native men are not conclusive and depend on the country of destination and the prevalence of obesity [15] of the home country [16,32], length of stay [31] or whether men are first or second generation in the host country [15,33]. For women, the highest prevalence of obesity among immigrants compared to natives is also consistent with previous studies in Europe regardless of the country of birth [14,15,16,26,32,34]. In addition, similarly to studies conducted in the Netherlands [16] and Sweden [14] with immigrants from Morocco, sub-Saharan Africa Central and South America, obesity inequalities between native and immigrant women, persist after adjusting for sociodemographic characteristics and health behaviors. Hence, place of birth in women may be considered a social determinant of obesity inequality. For women, factors associated with double discrimination, gender and immigration status, can explain their greater prevalence of obesity.

Despite the extant evidence on the protective effect of higher educational attainment on health status and specifically in obesity [18,19,21,22], we found a protective effect at lower educational levels in immigrant men relative to their native peers. This finding may be explained by the following reason: the health status before and after the migration process may help explain the different role of education on obesity for immigrant men. Before the migration process, evidence indicates that, even in their country of origin, the health status of immigrants is better than that of the rest of the population, a phenomenon known as healthy migrant effect [35]. Once in the host country, in this case Spain, regardless of education, immigrants are inserted in a precarious labor market segmented by place of birth and gender [36]. For immigrant men, the labor market includes construction, catering and agriculture requiring great physical effort and jobs in which discrimination attributable to obesity may be present [37]. This social reality may contribute to the lower prevalence of obesity among immigrant men with lower education, and thus, less chance to move into the labor market [38]. However, among immigrants with higher education, there is a mismatch between education and employment opportunities. This mismatch is referred in the literature as a determinant of health [39] and may explain the lack of a protective effect from education against obesity as we found in this study.

For immigrant women, as with immigrant men, the migration process is also triggered among those who are healthy [35]. However, in the host country, i.e., Spain, with the gender segmentation of the labor market, immigrant women are more likely to mainly hold domestic and care jobs, regardless of educational attainment [40]. This labor market is characterized by low-income, intense and long working hours, social isolation and poor opportunities for labor mobility leading to little control over schedules and to high level of stress [41]. The latter concurs with Sliwa et al. [42] findings of higher prevalence of obesity in immigrant women performing domestic work where strong physical demands are required. In addition, and as with immigrant men, the labor over-qualifications for immigrant women may explain the lack of a protective effect of education against obesity compared with native women. Thus, place of birth may affect how education related to health status among the immigrant population.

The study presents few limitations that deserve attention. First, this is a cross-sectional study and does not allow to establish a temporal relationship between exposure and outcome. Second, we could not present information according to country of birth for the immigrant population. The lack of this information may have masked greater obesity inequalities between the immigrant and the native populations. However, we repeated the analyses using region of birth (Europe, Latin America, Africa and Asia). Although not statistically significant, the results were consistent to the ones reported here for men (lower PR for immigrant relative to native) and women (higher PR for immigrant than native) with the exception of Asian women who exhibited lower prevalence of obesity than their native counterparts. Third, BMI was calculated using self-reported weight and height by the respondents. Evidence suggests that women may underestimate the body weight while men may overestimate their height. This misreporting may have biased the actual prevalence of obesity [43]. Finally, the proportion of missing values for BMI was higher for immigrants, with low education, especially in women. The latter may have underestimated our findings.

Despite the limitations, it is worth noting that this is the first study to examine obesity inequalities among a representative population of immigrants and natives in Spain. In addition, analyses were performed separately in women and in men to examine gender inequalities according to place of birth and further by educational attainment. Finally, we have access to a wide range of information collected through the surveys and a large sample size providing the power to examine interactions.

## 5. Conclusions

This study contributes to an understudied area, the investigation of obesity inequalities between immigrant and native populations. In addition, this study goes a step further by examining the joint effect of place of birth and education attainment. Our findings suggest that place of birth may affect obesity in both, women and men. However, this effect may be compounded with education differently for women and men. Thus, studies examining health outcome among immigrant populations relative to native populations should consider the inclusion of education as a potential partner for the effect of place of birth. A better understanding of how these factors work together is needed when designing strategies for health promotion to reduce obesity inequalities between immigrant and native populations regardless of gender. Furthermore, it is essential to develop gender-specific policies to promote equal opportunities in the labor market, especially among immigrant women with limited capacity to negotiate their working conditions.

## Figures and Tables

**Table 1:** Distribution of sociodemographic, self-rated health and health behavior characteristics for participants of the Spanish National Health Survey 2011–2012 and European Health Survey in Spain 2014.

Characteristic	Women				Men			
	Immigrants *n* = 1338	Natives *n* = 12,624	Total *n* = 13,962	*p*-Value *	Immigrants *n* = 1093	Natives *n* = 12,665	Total *n* = 13,758	*p*-Value *
	% (SE) **	% (SE)	% (SE)		% (SE)	% (SE)	% (SE)	
**Survey year**				<0.001				<0.001
2011	50.6 (1.7)	49.4 (0.5)	49.6 (0.5)		52.7 (1.9)	49.6 (0.5)	49.9 (0.5)	
2014	49.4 (1.7)	50.6 (0.5)	50.6 (0.5)		47.3 (1.9)	50.4 (0.5)	50.1 (0.5)	
**Region of origin**								
Europe	24.3 (1.5)				22.7 (1.6)			
Africa	16.2 (1.4)				27.0 (1.7)			
Latin America	54.2 (1.7)				44.8 (1.9)			
Asia	5.3 (0.8)				5.5 (0.9)			
**Age (years)**	36.8 (0.35)	41.9 (0.14)	41.2 (0.13)	<0.001	36.8 (0.42)	41.7 (0.14)	41.1 (0.13)	<0.001
18–24	11.3 (1.2)	10.6 (0.4)	10.7 (0.4)	<0.001	13.8 (1.4)	10.3 (0.4)	10.7 (0.4)	<0.001
25–44	65.2 (1.6)	45.4 (0.5)	48.3 (0.5)		62.4 (1.9)	46.8 (0.5)	48.7 (0.5)	
45–64	23.4 (1.4)	44.0 (0.5)	41.0 (0.5)		23.8 (1.6)	42.9 (0.5)	40.6 (0.5)	
**Educational attainment**				<0.001				<0.001
Primary or less	18.3 (1.4)	13.7 (0.4)	14.4 (0.4)		18.6 (1.5)	14.5 (0.4)	15.0 (0.4)	
Secondary	67.8 (1.6)	61.1 (0.5)	62.0 (0.5)		69.0 (1.7)	66.5 (0.5)	66.8 (0.5)	
Graduate or higher	13.9 (1.1)	25.3 (0.5)	23.6 (0.4)		12.3 (1.1)	19.1 (0.4)	18.2 (0.4)	
**Social Class**				<0.001				<0.001
Manual	83.1 (1.2)	55.9 (0.5)	59.8 (0.5)		84.0 (1.4)	58.4 (0.5)	61.4 (0.5)	
Non-manual	16.9 (1.2)	44.1 (0.5)	40.2 (0.5)		16.0 (1.4)	41.6 (0.5)	38.6 (0.5)	
**Employment status**				<0.001				<0.001
Employed	52.4 (1.7)	54.4 (0.5)	54.1 (0.5)		56.6 (1.9)	66.4 (0.5)	65.3 (0.5)	
Unemployed	22.8 (1.4)	17.2 (0.4)	18.0 (0.4)		33.5 (1.8)	18.2 (0.4)	20.0 (0.4)	
Others	24.7 (1.5)	28.5 (0.5)	27.9 (0.5)		9.9 (1.2)	15.4 (0.4)	14.7 (0.4)	
**Living arrangement**				<0.001				<0.001
Married/Couple	43.7 (1.7)	41.0 (0.5)	41.4 (0.5)		61.7 (1.8)	58.0 (0.5)	58.4 (0.5)	
Other	56.3 (1.7)	59.0 (0.5)	58.6 (0.5)		38.3 (1.8)	42.0 (0.5)	41.6 (0.5)	
**Self-rated health**				<0.001				<0.001
Good	71.1 (1.6)	76.0 (0.4)	75.3 (0.4)		81.1 (1.5)	81.2 (0.4)	81.2 (0.4)	
Poor	28.9 (1.6)	24.0 (0.4)	24.7 (0.4)		18.9 (1.5)	18.8 (0.4)	18.8 (0.4)	
**Smoking status**				<0.001				<0.001
Current Smoker	17.0 (1.2)	29.4 (0.5)	27.6 (0.5)		32.4 (1.5)	35.9 (0.5)	35.5 (0.5)	
Former Smoker	10.8 (1.0)	21.2 (0.4)	19.7 (0.4)		18.7 (1.5)	26.1 (0.5)	25.2 (0.4)	
Never smoked	72.2 (1.5)	49.5 (0.5)	52.7 (0.5)		48.9 (1.9)	38.0 (0.5)	39.3 (0.5)	
**Alcohol consumption**				<0.001				<0.001
Frequent	16.9 (1.2)	28.6 (0.5)	26.9 (0.4)		36.5 (1.8)	55.4 (0.5)	53.2 (0.5)	
Occasional	36.8 (1.6)	36.2 (0.5)	36.3 (0.5)		28.8 (1.7)	28.3 (0.5)	28.3 (0.5)	
Not last year/never	46.3 (1.7)	35.2 (0.5)	36.8 (0.5)		34.7 (1.8)	16.3 (0.4)	18.5 (0.4)	
**Workplace physical activity**				<0.001				<0.001
Sedentary	77.6 (1.4)	85.8 (0.4)	84.6 (0.4)		68.0 (1.7)	76.6 (0.4)	75.6 (0.4)	
Active	22.4 (14)	14.2 (0.4)	15.4 (0.4)		32.0 (1.7)	23.4 (0.4)	24.4 (0.4)	
**Leisure-time physical activity**				<0.001				<0.001
Sedentary	54.4 (1.7)	41.1 (0.5)	43.0 (0.5)		40.5 (1.9)	33.2 (0.5)	34.0 (0.5)	
Active	45.6 (1.7)	58.9 (0.5)	570 (0.5)		59.5 (1.9)	66.8 (0.5)	66.0 (0.5)	
**Daily Consumption of fruit and vegetable**				<0.001				<0.001
Yes	37.5 (1.7)	39.6 (0.5)	39.3 (0.5)		29.2 (1.7)	28.8 (0.5)	28.8 (0.5)	
No	62.5 (1.7)	60.4 (0.5)	60.7 (0.5)		70.8 (1.7)	71.2 (0.5)	71.2 (0.5)	
**BMI, Kg/M^2^**	25.7 (0.18)	24.5 (0.05)	24.7 (0.05)	<0.001	25.9 (0.13)	26.4 (0.04)	26.3 (0.04)	<0.001

* *p*-values from Chi-square statistics and *t*-tests. ** Means and SEs presented for age and BMI.

**Table 2:** Prevalence estimates for obesity according to place of birth in women and in men: Spanish National Health Survey 2011–2012 and European Health Survey in Spain 2014.

Characteristic	Women				Men			
	Immigrants *n* = 1338	Natives *n* = 12,624	Total *n* = 13,962	*p*-Value *	Immigrants *n* = 1093	Natives *n* = 12,665	Total *n* = 13,758	*p*-Value *
	% (SE)	% (SE)	% (SE)		% (SE)	% (SE)	% (SE)	
**Overall**	20.0 (1.4)	12.5 (0.3)	13.6 (0.4)	<0.001	12.5 (1.2)	16.9 (0.4)	16.4 (0.4)	<0.001
**Survey year**				0.257				0.192
2011	16.3 (1.9)	12.7 (0.5)	13.2 (0.5)		12.9 (1.7)	17.4 (0.6)	16.9 (0.5)	
2014	23.8 (2.1)	12.3 (0.5)	14.0 (0.5)		12.0 (2.1)	16.5 (0.5)	16.0 (0.5)	
**Region of origin**								
Europe	15.4 (2.8)				13.4 (2.6)			
Africa	30.9 (4.3)				10.7 (2.3)			
Latin America	20.5 (1.9)				13.8 (1.9)			
Asia	2.7 (1.7)				6.1 (3.4)			
**Age (years)**				<0.001				<0.001
18–24	6.4 (2.5)	4.4 (0.8)	4.7 (0.8)		2.8 (1.5)	5.4 (0.9)	5.0 (0.8)	
25–44	18.4 (1.7)	9.7 (0.5)	11.4 (0.5)		12.4 (1.5)	13.7 (0.5)	13.5 (0.5)	
45–64	30.9 (3.4)	17.3 (0.6)	18.4 (0.6)		18.4 (3.0)	23.2 (0.6)	22.9 (0.6)	
**Educational attainment**				<0.001				<0.001
Primary or less	35.2 (4.4)	25.6 (1.2)	27.4 (1.3)		10.3 (2.8)	24.8 (1.2)	22.7 (1.1)	
Secondary	17.5 (1.6)	12.3 (0.4)	13.2 (0.5)		12.5 (1.5)	17.1 (0.5)	16.5 (0.5)	
Graduate or higher	12.1(2.7)	5.8 (0.5)	6.3 (0.5)		15.5 (3.4)	10.5 (0.7)	10.9 (0.7)	
**Social class**				<0.001				<0.001
Manual	21.7 (1.6)	15.7 (0.5)	16.9 (0.5)		11.8 (1.3)	19.5 (0.5)	18.3 (0.5)	
Non-manual	11.4 (2.5)	8.4 (0.4)	8.6 (0.4)		16.0 (3.2)	13.3 (0.5)	13.5 (0.5)	
**Employment status**				<0.001				0.016
Employed	16.1 (1.8)	9.4 (0.4)	10.3 (0.4)		12.1 (1.5)	16.2 (0.5)	15.7 (0.4)	
Unemployed	22.1 (2.9)	16.0 (1.0)	17.1 (0.9)		15.2 (2.5)	18.9 (1.0)	18.2 (0.9)	
Others	26.2 (3.4)	16.3(0.7)	17.6 (0.8)		5.3 (2.5)	18.0 (1.0)	17.0 (1.0)	
**Living arrangement**				<0.001				<0.001
Married/Couple	23.4 (2.1)	14.2 (0.5)	15.5 (0.5)		16.7 (1.8)	20.0 (0.5)	19.5 (0.5)	
Other	15.6 (1.9)	10.0 (0.5)	10.9 (0.5)		5.6 (1.1)	12.8 (0.5)	12.0 (0.5)	
**Self-rated health**				<0.001				<0.001
Good	14.8 (1.5)	9.6 (0.4)	10.3 (0.4)		11.9 (1.3)	14.9 (0.4)	14.5 (0.4)	
Poor	32.7 (3.2)	21.6 (0.9)	23.5 (0.9)		14.9 (1.3)	26.0 (1.0)	24.6 (1.0)	
**Smoking status**				0.032				<0.001
Current smoker	21.0 (3.5)	10.9 (0.6)	11.8 (0.6)		9.2 (1.9)	15.5 (0.6)	14.8 (0.6)	
Former smoker	22.0 (3.9)	12.4 (0.8)	13.1 (0.8)		17.3 (3.2)	23.2 (0.8)	22.7 (0.8)	
Never smoked	19.4 (1.7)	13.5 (0.5)	14.7 (0.5)		12.8 (1.8)	14.0 (0.6)	13.8 (0.6)	
**Alcohol consumption**				<0.001				0.110
Frequent	14.0 (2.7)	8.8 (0.5)	9.2 (0.6)		13.8 (2.1)	16.4 (0.5)	16.2 (0.5)	
Occasional	17.5 (2.2)	11.2 (0.5)	12.2 (0.6)		14.2 (2.5)	16.3 (0.7)	16.1 (0.7)	
Not last year/never	24.1 (2.3)	16.8 (0.7)	18.1 (0.7)		9.7 (1.8)	20.0 (1.1)	17.7 (0.9)	
**Workplace physical activity**				0.718				0.273
Sedentary	19.7 (1.6)	12.5 (0.4)	13.4 (0.4)		12.8 (1.5)	17.2 (0.4)	16.7 (0.4)	
Active	20.9 (3.1)	12.7 (0.9)	14.4 (1.0)		11.7 (2.2)	16.2 (0.8)	15.5 (0.7)	
**Leisure-time physical activity**				<0.001				<0.001
Sedentary	22.8 (2.1)	16.0 (0.6)	17.2 (0.6)		12.2 (1.8)	24.0 (0.8)	22.4 (0.7)	
Active	16.7 (1.9)	10.1 (0.4)	10.8 (0.4)		12.7 (1.6)	13.4 (0.4)	13.3 (0.4)	
**Daily Consumption of fruit and vegetables**				0.409				0.471
Yes	19.8 (2.4)	13.0 (0.5)	13.4 (0.5)		12.5 (1.2)	17.4 (0.7)	16.8 (0.7)	
No	20.1 (1.8)	12.2 (0.4)	13.9 (0.6)		12.4 (1.5)	16.8 (0.5)	16.2 (0.4)	

* *p*-values from Cochran-Mantel-Haenszel statistics.

**Table 3:** Prevalence ratios and their 95% confidence intervals of obesity for place of birth in women and in men: Spanish National Health Survey 2011–2012 and European Health Survey in Spain 2014.

Place of Birth	Unadjusted	Model 1 *	Model 2	Model 3	Model 4	Model 5
**Women**						
Natives	1.00	1.00	1.00	1.00	1.00	1.00
Immigrants	1.60 (1.3–1.86)	1.60 (1.3–1.86)	1.73 (1.5–2.00)	1.73 (1.5–2.01)	1.56 (1.3–1.81)	1.42 (1.2–1.64)
**Men**						
Natives	1.00	1.00	1.00	1.00	1.00	1.00
Immigrants	0.74 (0.60–0.89)	0.73 (0.6–0.89)	0.83 (0.6–1.01)	0.79 (0.6–0.95)	0.75 (0.6–0.92)	0.73 (0.5–0.89)

* Model 1 adjusted for survey year; model 2 additionally adjusted for age and self-rated health; model 3 additionally adjusted for employment status and living arrangement; model 4 additionally adjusted for physical activity at work and at leisure time, smoking, alcohol consumption and daily consumption of fruits and vegetables; and model 5 additionally adjusted for social class and education attainment.
